# The Dynamic Immunological Parameter Landscape in Coronavirus Disease 2019 Patients With Different Outcomes

**DOI:** 10.3389/fimmu.2021.697622

**Published:** 2021-10-29

**Authors:** Guoxing Tang, Min Huang, Ying Luo, Wei Liu, Qun Lin, Liyan Mao, Shiji Wu, Zhigang Xiong, Hongyan Hou, Ziyong Sun, Feng Wang

**Affiliations:** Department of Laboratory Medicine, Tongji Hospital, Tongji Medical College, Huazhong University of Science and Technology, Wuhan, China

**Keywords:** COVID-19, innate immunity, adaptive immunity, humoral immunity, outcome

## Abstract

**Objectives:**

The longitudinal and systematic evaluation of immunity in coronavirus disease 2019 (COVID-19) patients is rarely reported.

**Methods:**

Parameters involved in innate, adaptive, and humoral immunity were continuously monitored in COVID-19 patients from onset of illness until 45 days after symptom onset.

**Results:**

This study enrolled 27 mild, 47 severe, and 46 deceased COVID-19 patients. Generally, deceased patients demonstrated a gradual increase of neutrophils and IL-6 but a decrease of lymphocytes and platelets after the onset of illness. Specifically, sustained low numbers of CD8^+^ T cells, NK cells, and dendritic cells were noted in deceased patients, while these cells gradually restored in mild and severe patients. Furthermore, deceased patients displayed a rapid increase of HLA-DR expression on CD4^+^ T cells in the early phase, but with a low level of overall CD45RO and HLA-DR expressions on CD4^+^ and CD8^+^ T cells, respectively. Notably, in the early phase, deceased patients showed a lower level of plasma cells and antigen-specific IgG, but higher expansion of CD16^+^CD14^+^ proinflammatory monocytes and HLA-DR^−^CD14^+^ monocytic-myeloid-derived suppressor cells (M-MDSCs) than mild or severe patients. Among these immunological parameters, M-MDSCs showed the best performance in predicting COVID-19 mortality, when using a cutoff value of ≥10%. Cluster analysis found a typical immunological pattern in deceased patients on day 9 after onset, which was characterized as the increase of inflammatory markers (M-MDSCs, neutrophils, CD16^+^CD14^+^ monocytes, and IL-6) but a decrease of host immunity markers.

**Conclusions:**

This study systemically characterizes the kinetics of immunity of COVID-19, highlighting the importance of immunity in patient prognosis.

## Introduction

Coronavirus disease 2019 (COVID-19), an infectious disease caused by the novel severe acute respiratory syndrome coronavirus 2 (SARS-CoV-2), has become the greatest threat to global public health (1–3). Globally, as of January 20, 2021, there have been 94.9 million confirmed cases of COVID-19, including 2.1 million deaths, as reported by the World Health Organization (4). Currently, there is still no confirmed effective therapeutic strategy for COVID-19, highlighting the importance of further understanding the pathogenesis of the disease.

In contrast to the decrease of peripheral blood CD4^+^ and CD8^+^ T-cell count (5), deceased patients demonstrated increased expressions of HLA-DR and CD38 on these cells, which emphasized the importance of overactivation of adaptive immunity in the pathogenesis of the disease (6). Consistent with this notion, severe patients showed a higher level of plasma cytokines such as IL-2, IL-6, TNF-α, and IL-10, compared to mild patients, supporting the evidence that high inflammatory status and dysregulation of immunity were involved in patients with poor outcome (7). In line with these findings, some studies have demonstrated that innate immune responses in patients with uncontrolled SARS-CoV-2 infection are exaggerated and remain elevated, contributing to tissue damage (8–10). In contrast, previous studies also have reported that old age, low CD8^+^ T cells, and underlying diseases are risk factors for COVID-19 progression, which indicates that patients with initial low immunity are more likely to develop severe disease (7, 11). We thus speculated that either hyperimmune or hypoimmune status could be noted in COVID-19 patients and that different immune statuses could predict the severity and mortality due to COVID-19.

Although many previous studies have focused on the characteristics of host immunity in COVID-19 patients with different severity (12–15), there were rare studies that systemically investigated the kinetics of immunity in patients with COVID-19. Here, we monitored the dynamics of immune responses, including innate, adaptive, and humoral immunity, in COVID-19 patients with different severity.

## Methods

### Patients

Between February 2020 and May 2020, patients with confirmed COVID-19 were recruited from Tongji Hospital, Wuhan, China; and the immune indicators involved in innate, adaptive, and humoral immunity were continuously determined in these patients. Confirmed COVID-19 was defined in patients who had clinical and radiological characteristics of COVID-19 and together with positive SARS-CoV-2 real-time RT-PCR results. The COVID-19 patients were classified into three groups according to different severity and outcome during 45 days after onset of illness: 1) mild group, patients have saturation of oxygen (SpO_2_) ≥94% on room air during hospitalization and finally discharge; 2) severe group, patients have sign of hypoxia (respiration rate ≥30 times/min, SpO_2_ ≤93%, or PaO_2_/FiO_2_ ratio ≤300 mmHg), recovered, and then discharges; and 3) deceased group, patients have respiratory failure requiring mechanical ventilation, septic shock, and/or multiple organ dysfunction and then died during hospitalization (16). Patients without a definite clinical outcome and remained in hospital until 45 days after onset of illness were excluded. Disease courses were classified into the early (0–15 days), middle (16–30 days), and late (31–45 days) phases based on days after onset of symptoms. Patients admitted during the study period received supportive and therapeutic modalities based on individual physician’s clinical discretion and our inpatient guide. The main treatments included supplemental oxygen, antibiotics, antiviral drug, corticosteroids, and intravenous immunoglobulin. The routine blood test and immunological monitoring (the number and phenotype of immune cells, cytokines, and SARS-CoV-2-specific IgG) were performed every 3 to 9 days until 45 days after onset of illness. The demographic and clinical information, laboratory results, and outcome data were collected from electronic medical records. All patients discharged from the hospital within 45 days were subsequently be transferred to isolation hotels, and blood samples were still collected until 45 days after onset of illness. This study was approved by the ethical committee of Tongji Hospital, Tongji Medical College, Huazhong University of Science and Technology, Wuhan, China (IRB ID: TJ-C20200128). Written informed consent was obtained from all the participants.

### Routine Blood Test, SARS-CoV-2-Specific IgG, and Cytokine Profile Analysis

Blood samples were collected from study participants. The absolute numbers of neutrophils, lymphocytes, monocytes, and platelets were measured by an automatic blood cell counter. The SARS-CoV-2-specific IgG, which targets the receptor-binding domain of the spike protein, was detected by paramagnetic particle chemiluminescent immunoassay using iFlash-SARS-CoV-2 IgG assay kit (YHLO Biotech Co., Ltd. Shenzhen, China). The levels of IL-2 receptor (IL-2R) and IL-10 in serum were measured according to an automatic procedure of a solid-phase two-site chemiluminescent immunometric assay *via* IMMULITE 1000 Analyzer (Siemens). The level of IL-6 was measured by the electrochemiluminescence method (Roche Diagnostics).

### Flow Cytometry Analysis

Heparinized peripheral blood was collected from study participants. Fluorescence-labeled monoclonal antibodies against the following antigens were added to the cell suspensions: CD45, CD3, CD4, CD8, CD45RA, CD45RO, HLA-DR, CD19, CD27, CD38, CD86, CD14, CD16, and CD56 (BD Biosciences). Isotype controls with irrelevant specificities were included as negative controls. All these cell suspensions were incubated for 30 min on ice. After washing, the pellets were resuspended in 300 μl of staining buffer, followed by analysis with FACSCanto flow cytometer (BD Biosciences). Gating strategies of HLA-DR^+^ CD4^+^ and CD8^+^ T cells, CD45RO^+^CD4^+^ T cells, CD27^+^CD38^−^ memory B cells, CD27^+^CD38^high^ plasma cells, CD16^+^CD14^+^ monocytes (non-classical monocytes), HLA-DR^−^CD14^+^ monocytic-myeloid-derived suppressor cells (M-MDSCs), lymphoid-derived dendritic cells (DCs), and CD86^+^ lymphoid-derived DCs are shown in **Supplementary Figures 1****–****4**. The definition and gating strategy of M-MDSCs were according to a previous study (17). The percentage of CD3^−^CD19^−^CD14^−^CD56^−^HLA-DR^+^ cells in CD45^+^ lymphocytes was defined as the percentage of lymphoid-derived DCs. The number of lymphoid-derived DCs was calculated by multiplying the percentage of lymphoid-derived DCs with total lymphocyte count.

### TBNK Lymphocyte Subset

The absolute numbers of CD4^+^ and CD8^+^ T cells, B cells, and NK cells were determined by using TruCOUNT tubes and BD Multitest 6-color TBNK Reagent Kit (BD Biosciences) according to the manufacturer’s instructions.

### Statistical Analysis

The results are presented as mean ± standard deviation (SD), or as median with interquartile range (IQR), when appropriate. Continuous variables were compared with one-way ANOVA test. Chi-square test was used for categorical data. To aid visualization, a smoothing spline was fitted to the values of different immunological parameters to summarize the overall trend. Receiver operating characteristic (ROC) curve analysis was performed on immune indicators to assess the cutoff values. The Kaplan–Meier survival analysis was used to analyze the cumulative survival, and a log-rank test was used to determine differences in survival. Hierarchical cluster analysis was performed to determine the typical immunological pattern among different groups of patients, and correlation between different immunological indicators was analyzed. Statistical significance was determined as *p* < 0.05 (*p < 0.05, **p < 0.01, ***p < 0.001). Statistical analyses were performed using SPSS version 19.0 (SPSS, Chicago, IL), GraphPad Prism 8.0 (San Diego, CA, USA), and R 4.0.3 (R Foundation, Vienna, Austria).

## Results

### Clinical Characteristics of Coronavirus Disease 19 Patients

The demographic and clinical characteristics of included patients are shown in **Table 1**. Ninety-three patients with COVID-19, including 47 severe and 46 deceased patients, were enrolled from Tongji Hospital. Another 27 mild COVID-19 patients were included and matched for age and gender to the severe group. No significant difference in age, gender, symptoms at the onset of illness, imaging features, and time from onset to admission was recorded among these three groups. Deceased patients showed a higher percentage of comorbidities such as chronic obstructive pulmonary disease and cardiovascular disease than did mild patients. Almost half of the patients had common treatment, including antibiotics, antiviral drugs, and corticosteroids. The proportion of patients treated with corticosteroids and intravenous immunoglobulin in the mild group was significantly lower than in the severe and deceased groups.

**Table 1:** The demographic and clinical characteristics of patients with COVID-19.

	① Mild (n = 27)	② Severe (n = 47)	③ Deceased (n = 46)	*p*-Value		
				① *vs.* ②	① *vs.* ③	② *vs.* ③
**Age, years, mean (SD)**	68.34 (10.71)	67.10 (11.46)	69.15 (15.75)	0.663	0.331	0.263
**Male sex**	18 (66.67)	31 (65.96)	30 (65.22)	0.951	0.899	0.940
**Symptoms at onset of illness**						
Fever	20 (74.07)	38 (80.85)	35 (76.09)	0.495	0.847	0.576
Cough	17 (62.96)	31 (65.96)	30 (65.22)	0.795	0.846	0.940
Shortness of breath	8 (29.63)	17 (36.17)	16 (34.78)	0.570	0.651	0.889
Chest distress	4 (14.81)	8 (17.02)	9 (19.57)	0.804	0.609	0.751
Fatigue	3 (11.11)	5 (10.64)	6 (13.04)	0.950	0.808	0.720
Expectoration	5 (18.52)	11 (23.40)	12 (26.09)	0.623	0.460	0.764
Diarrhea	4 (14.81)	8 (17.02)	8 (17.39)	0.804	0.774	0.962
Headache	3 (11.11)	7 (14.89)	7 (15.22)	0.647	0.622	0.965
Muscle ache	2 (7.41)	3 (6.38)	3 (6.52)	0.866	0.885	0.978
Pharyngalgia	1 (3.70)	2 (4.26)	2 (4.35)	0.908	0.894	0.983
Nausea and vomiting	2 (7.41)	2 (4.25)	2 (4.35)	0.564	0.579	0.983
**Comorbidities**						
Hypertension	13 (48.15)	25 (53.19)	22 (47.83)	0.676	0.979	0.605
Diabetes	4 (14.81)	11 (23.40)	12 (26.09)	0.376	0.261	0.764
Cardiovascular disease	2 (7.41)	9 (19.15)	13 (28.26)	0.172	0.033	0.301
Malignancy	2 (7.41)	4 (8.51)	5 (10.87)	0.867	0.628	0.701
Cerebrovascular disease	1 (3.70)	3 (6.38)	3 (6.52)	0.624	0.670	0.978
Chronic kidney disease	1 (3.70)	2 (4.26)	2 (4.35)	0.908	0.894	0.983
Transplantation	0 (0)	2 (4.26)	2 (4.35)	0.277	0.272	0.983
COPD	0 (0)	3 (6.38)	7 (15.22)	0.180	0.033	0.169
Tuberculosis history	0 (0)	2 (4.26)	2 (4.35)	0.277	0.272	0.983
Chronic liver disease	1 (3.70)	1 (2.13)	2 (4.35)	0.687	0.894	0.545
**Imaging features**						
Unilateral pneumonia	2 (7.41)	3 (6.38)	2 (4.35)	0.866	0.579	0.664
Bilateral pneumonia	23 (85.19)	44 (93.61)	41 (89.13)	0.233	0.621	0.440
Ground-glass opacity	5 (18.52)	9 (19.15)	10 (21.74)	0.947	0.742	0.759
**Treatment**						
Antibiotics	11 (40.74)	25 (53.19)	27 (58.70)	0.302	0.138	0.593
Antiviral treatment	15 (55.56)	24 (51.06)	25 (54.35)	0.710	0.920	0.751
Corticosteroids	9 (33.33)	27 (57.45)	19 (41.30)	0.046	0.498	0.120
Intravenous immunoglobulin	5 (18.52)	18 (38.30)	20 (43.48)	0.077	0.030	0.611
**Days from onset to admission, median (range)**	9 (3–24)	12 (3–24)	9 (3–21)	0.992	0.931	0.899
**Days from onset to discharge or death, median (range)**	29 (18–41)	35 (25–45)	29 (15–45)	0.032	NA	NA
**ICU admission**	0 (0)	3 (6.38)	46 (100)	0.180	<0.001	<0.001
**Length of ICU stay, days, median (range)**	0 (0–0)	9 (5–14)	9 (4–24)	NA	NA	NA

Data are presented as numbers (%) unless otherwise indicated.

COVID-19, coronavirus disease 2019; COPD, chronic obstructive pulmonary disease; ICU, intensive care unit; SD, standard deviation; NA, not applicable.

### Routine Blood Test and Cytokine Results of Coronavirus Disease 2019 Patients

**Figure 1:**
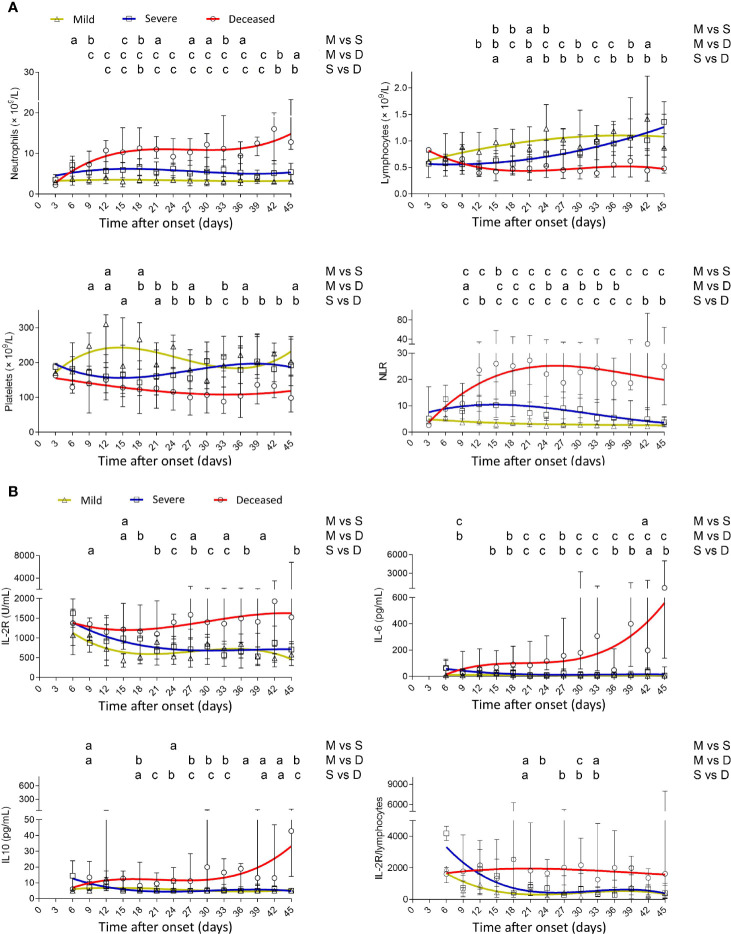
Dynamic analysis of routine blood test and cytokines in patients with different outcomes. **(A)** The absolute numbers of neutrophils, lymphocytes, platelets, and NLR and **(B)** the values of IL-2R, IL-6, IL-10, and IL-2R/lymphocytes in the peripheral blood of mild (yellow line), severe (blue line), and deceased (red line) COVID-19 patients were analyzed at different time points (every 3 days from day 3 to day 45 after onset of illness). Data in each time point are expressed as median with interquartile range. The smoothing cubic splines were fitted to the values of different parameters to summarize the overall trend. NLR, neutrophil-to-lymphocyte ratio; IL-2R, IL-2 receptor. ^a^*p* < 0.05; ^b^*p* < 0.01; ^c^*p* < 0.001. M, mild; S, severe; D, deceased.

A sustained lower level of neutrophils was observed in mild patients compared with severe or deceased patients. Conversely, neutrophils gradually increased after onset, reached a maximum at 3 weeks, and were maintained at a high level after that in deceased patients. Deceased patients showed a gradual loss of lymphocytes after onset, but lymphocytes in mild and severe patients gradually restored to the normal range. Thus, deceased patients displayed a rapid increase of neutrophil-to-lymphocyte ratio (NLR) in the early phase of the disease. Platelets in deceased patients also gradually decreased after onset, but in severe patients, they were gradually restored after a transient decline (**Figure 1A**). IL-2R in deceased patients remained at a high level during the illness, while in mild and severe patients, it gradually declined after onset. Both IL-6 and IL-10 had an increased trend in deceased patients with the progress of the disease but stayed at a low level in mild and severe patients. Similar to NLR, IL-2R/lymphocytes in deceased patients remained at a high level during the illness (**Figure 1B**).

### The Number of Different Subsets of Lymphocytes, Lymphoid-Derived Dendritic Cells, and Monocytes in COVID-19 Patients

**Figure 2:**
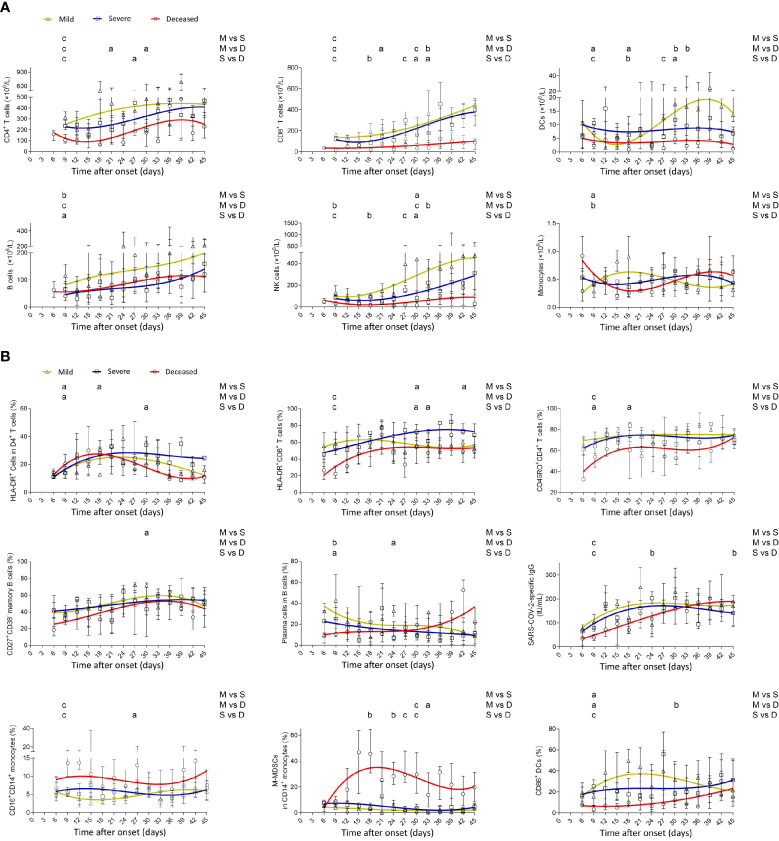
Dynamic analysis of the number, phenotype, and function of immune cells in patients with different outcomes. **(A)** The absolute numbers of CD4^+^ T cells, CD8^+^ T cells, B cells, NK cells, lymphoid-derived DCs, and monocytes and **(B)** the percentages of HLA-DR^+^CD4^+^ T cells, HLA-DR^+^CD8^+^ T cells, CD45RO^+^CD4^+^ T cells, CD27^+^CD38^−^ memory B cells within CD19^+^ B cells, CD27^+^CD38^high^ plasma cells within CD19^+^ B cells, SARS-CoV-2-specific IgG, CD16^+^CD14^+^ monocytes, HLA-DR^−^CD14^+^ M-MDSCs within monocytes, and CD86^+^ lymphoid-derived DCs in the peripheral blood of mild (yellow line), severe (blue line), and deceased (red line) COVID-19 patients were analyzed at different time points (every 3 days from day 3 to day 45 after onset of illness). Data in each time point are expressed as median with interquartile range. The smoothing cubic splines were fitted to the values of different parameters to summarize the overall trend. Lymphoid-derived DCs, dendritic cells; SARS-CoV-2, severe acute respiratory syndrome coronavirus 2; M-MDSCs, monocytic-myeloid-derived suppressor cells. ^a^*p* < 0.05; ^b^*p* < 0.01; ^c^*p* < 0.001. M, mild; S, severe; D, deceased.

Generally, deceased patients displayed a lower number of different subsets of lymphocytes, including CD4^+^ and CD8^+^ T cells, B cells, and NK cells, than did mild patients. Notably, the numbers of CD8^+^ T cells and NK cells in deceased patients were maintained at a low level during the illness, whereas those in mild and severe patients were gradually restored with the development of the disease. Consistent with NK cells, lymphoid-derived DCs in deceased patients remained at a low level during the illness. In contrast to the gradual increase of monocytes in mild patients, the number of monocytes in deceased patients gradually decreased in the early phase but gradually increased from the middle to late phase of the disease (**Figure 2A**).

### The Phenotype and Function of Immune Cells in COVID-19 Patients

HLA-DR expression on CD4^+^ T cells showed a rapid increase in the early phase in deceased patients and then gradually decreased in the late phase. Although CD45RO expression on CD4^+^ T cells and HLA-DR expression on CD8^+^ T cells had an increased trend in deceased patients after onset, their overall levels in deceased patients were lower than in mild patients, especially in the early phase. The frequency of plasma cells within B cells in mild patients rapidly reached maximum after onset, while in deceased patients, it had a slow increased trend during the illness. Consistent with plasma cells, SARS-CoV-2-specific IgG took a long time to reach maximum in deceased patients compared with mild or severe patients. Notably, deceased patients demonstrated higher percentages of CD16^+^CD14^+^ non-classical proinflammatory monocytes and HLA-DR^−^CD14^+^ M-MDSCs compared with both mild and severe patients. The overall expression of CD86 on lymphoid-derived DCs in deceased patients was lower than that in mild and severe patients (**Figure 2B**).

### Using Immunological Parameters for Predicting COVID-19 Mortality

**Figure 3:**
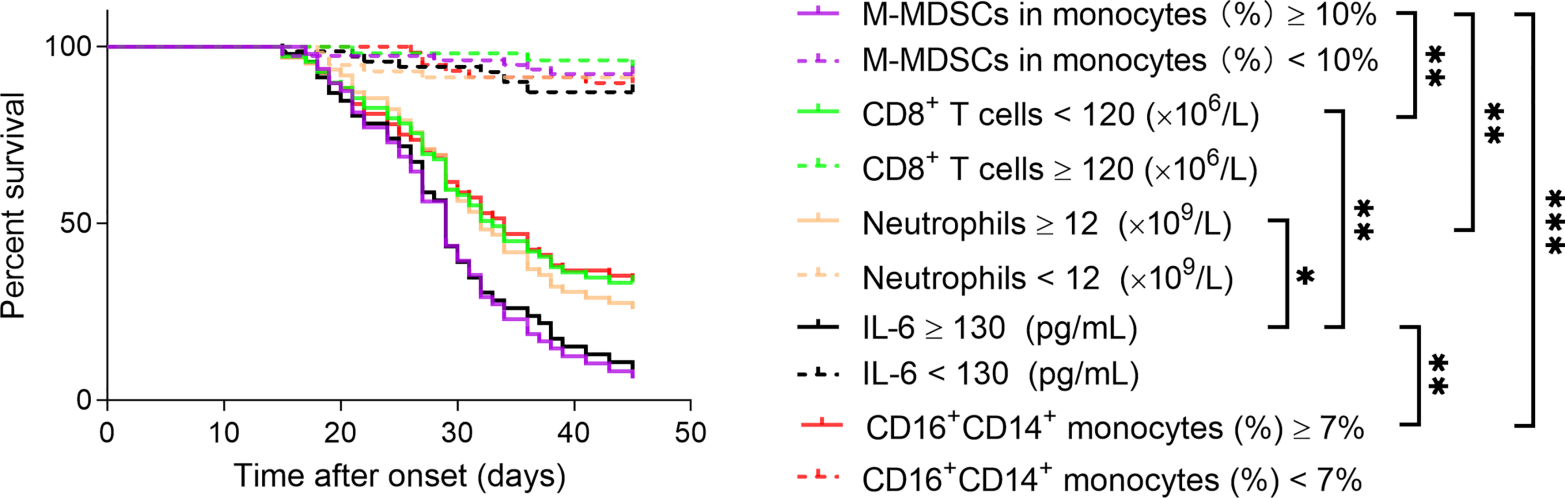
Using immune parameters for predicting COVID-19 mortality. The effect of immune parameters including M-MDSCs, CD8^+^ T cells, neutrophils, IL-6, and CD16^+^CD14^+^ monocytes on predicting 45-day mortality after onset of illness was analyzed in COVID-19 patients. M-MDSCs, monocytic-myeloid-derived suppressor cells. *p < 0.05, **p < 0.01, ***p < 0.001.

The Kaplan–Meier method was further used to analyze the cumulative survival of patients and the effect of these immunological parameters on survival. The cutoff values of these immune parameters used for survival analysis were determined by ROC analysis (the maximum of M-MDSCs, neutrophils, IL-6, and CD16^+^CD14^+^ monocytes and the minimum of CD8^+^ T cells in different time points were used for each patient). We found that CD8^+^ T cells were positively correlated with survival rate; but M-MDSCs, neutrophils, IL-6, and CD16^+^CD14^+^ monocytes were conversely negatively correlated with survival rate in enrolled patients. Among these immune parameters, M-MDSCs showed the best performance in predicting COVID-19 mortality, when using cutoff value of ≥10% (**Figure 3**). ROC analyses of these markers for distinguishing deceased patients from other patients are shown in **Supplementary Figure 5**.

### Clustering Analysis of Immunological Parameters in COVID-19 Patients on Day 9 After Onset

**Figure 4:**
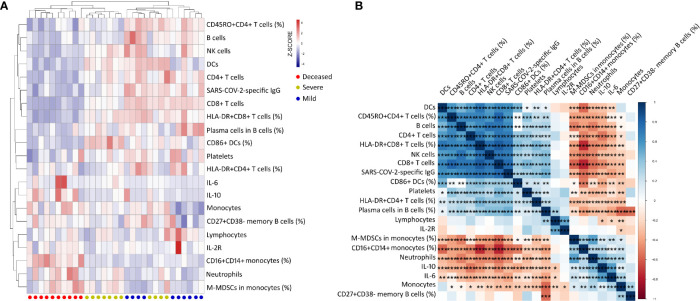
Clustering analysis of immune parameters in COVID-19 patients with different outcomes on day 9 after onset of illness. **(A)** Heat maps comparing 21 immune parameters in mild (n = 10), severe (n = 11), and deceased (n = 10) patients. On the y-axis are immune parameter values after z-scoring, and on the x-axis are individual patients. Red–white–blue squares represent z-scoring values. Red dot, deceased patient; yellow dot, severe patient; blue dot, mild patient. **(B)** Correlation matrix of 21 immune parameters in 31 COVID-19 patients (mild, n = 10; severe, n = 11; deceased, n = 10). M-MDSCs, monocytic-myeloid-derived suppressor cells; IL-2R, IL-2 receptor; lymphoid-derived DCs, dendritic cells. *p < 0.05, **p < 0.01, ***p < 0.001.

To determine the effect of immunological parameters on predicting disease outcome in the early phase, the immunological data were compared between different groups of patients on day 9 after onset. Nevertheless, samples were collected from only a small number of patients (mild group, n = 10; severe group, n = 11; deceased group, n = 10). Many immunological indicators, such as neutrophils, IL-6, CD4^+^ and CD8^+^ T cells, B cells, lymphoid-derived DCs, HLA-DR^+^CD8^+^ T cells, CD16^+^CD14^+^ monocytes, and M-MDSCs, had significant differences among these three groups (**Supplementary Table 1**). Subsequent hierarchical cluster analysis showed that these immunological indicators could better distinguish mild, severe, and deceased patients. In comparison with mild and severe patients, deceased patients displayed a typical immunological pattern in the early phase of disease, characterized as the increase of inflammatory markers including monocytes, CD16^+^CD14^+^ monocytes, neutrophils, M-MDSCs, IL-6, and IL-10, and the decrease of host immunity markers including CD4^+^ T cells, CD8^+^ T cells, B cells, NK cells, lymphoid-derived DCs, platelets, HLA-DR^+^CD8^+^ T cells, and CD45RO^+^CD4^+^ T cells (**Figure 4A**). Further correlation analysis demonstrated that these inflammatory markers were obviously negatively correlated with host immunity markers in COVID-19 patients with different outcomes (**Figure 4B**).

## Discussion

Increasing studies have indicated that one of the biggest drivers of COVID-19 mortality is cytokine storm (7, 18, 19). This potentially deadly condition can cause acute respiratory distress syndrome (ARDS) and multiple organ failure, which are two of the primary causes of mortality in severe COVID-19 patients (20–25). As a result, the dysregulation of immune cells, especially the lymphocytes and monocytes, is closely involved with the pathogenesis of severe COVID-19. Recently, the overall view of peripheral blood immune cells in COVID-19 patients remains obscure. This study has attempted to elucidate the dynamic immunological parameter landscape, including both the number and function of innate (neutrophils, NK cells, monocytes, and lymphoid-derived DCs), adaptive (CD4^+^ and CD8^+^ T cells), and humoral (B cells, plasma cells, and antigen-specific IgG) immune cells, in COVID-19 patients with different outcomes.

Previous studies have focused on the characteristics of host immunity in COVID-19 patients. However, most studies determined this only at a single time point, such as on admission or before discharge (11, 26–32). Furthermore, many studies did not classify the patients according to time after onset, age, or underlying diseases (11, 33). Our previous studies have confirmed that age can affect the number and phenotype of immune cells (34, 35). More specifically, in contrast to the gradual increase of HLA-DR expression on CD4^+^ and CD8^+^ T cells, the number of CD4^+^ and CD8^+^ T cells gradually decreased with increasing age (35, 36). Thus, understanding the conclusions of immunological data should be done with caution if these studies did not balance patients according to different influencing factors, and some data could be misleading to some extent. In view of these shortcomings, the present study has made some improvements. First, this is a longitudinal study, and the immunological data were continuously monitored from onset of illness to 45 days after onset. Second, in case of bias, many influencing factors such as age and gender were matched between different groups when including patients. Third, to elucidate the pathogenesis of the disease, we attempted to simultaneously investigate as many immunological parameters as we could, involving neutrophils, lymphocytes, monocytes, platelets, T cells, B cells, NK cells, lymphoid-derived DCs, plasma cells, M-MDSCs, cytokines, and SARS-CoV-2-specific antibody, which is caused by our comprehensive immune monitoring program in clinical practice.

Many previous studies have focused on the kinetics of immunity in COVID-19 patients with different severity (27, 37–40). Lucas et al. demonstrated that a concomitant reduction in T-cell number and an early elevation in cytokine levels were associated with worse disease outcomes (40), which are in accordance with our findings. Furthermore, a previous study has shown that a subgroup of patients had T-cell activation characteristic of acute viral infection and plasmablast responses reaching >30% of circulating B cells (37), which is consistent with our experimental results. However, a previous study has also demonstrated that more severe cases have a late onset in the humoral response as compared with mild/moderate infections (39), which is in line with our recent observations showing that the differentiation of plasma cells was delayed in severe patients. Regarding monocytes, consistent with previous findings, we also observed the CD16^+^ non-classical monocytes increased early with a reduction in classical CD16^−^ monocytes in severe patients (38). Nevertheless, the innovation of this study is that we investigated the dynamics of innate, adaptive, and humoral responses simultaneously in COVID-19 patients with different severity. A previous study has shown that the differentiation of plasma cells is quicker in mild patients compared with severe patients (39). Thus, the percentage of plasma cells in mild patients may have reached the peak at detection and then gradually decreased over time. However, some long-live memory plasma cells could persistently secrete a high level of antibodies, which could be the possible cause of the discrepancy in the percentage of plasma cells and antibody level in mild patients. There is no doubt that host immunity will gradually develop into a hypo or even anergic state in the late phase of deceased COVID-19 patients. However, identifying the immune characteristics in the early phase of the disease is the key to predicting disease prognosis. Based on the findings of this study, the following immune characteristics should be mentioned in the early phase of COVID-19: 1) increase of neutrophils but accompanying decrease of lymphocytes, leading to a rapid increase of NLR, is the most distinctive feature noted in the peripheral blood of deceased patients; 2) decrease of T-cell number, with low expression of functional markers such as HLA-DR and CD45RO, predicts the high risk of severe disease, which highlights the importance of adaptive immunity in controlling SARS-CoV-2 infection; 3) low level of lymphoid-derived DCs and NK cells, with decreased expression of CD86 on lymphoid-derived DCs, indicates a low innate immunity in patients with poor outcome; 4) low level of plasma cells and SARS-CoV-2-specific IgG supports the idea that delayed differentiation of plasma cells and antibody production are the typical characteristics of humoral immunity in patients with poor outcome; 5) increase of non-classical monocytes and IL-6, suggesting a high inflammatory state, is an important factor for predicting poor outcome; and 6) a rapid increase of inhibitory cells, especially the sustained expansion of M-MDSCs, can further aggravate immune deficiency, which could be the key factor to cause persistent low immunity in patients with poor outcome. Importantly, in the early phase of the disease, SARS-CoV-2-infected patients who have low innate, adaptive, and humoral immunity, but with high inflammatory status, tend to develop more severe disease. These findings confirm that the initial hypoimmune status is the most important risk factor for the poor outcome of the disease.

Given that cytokine storm is one of the leading causes of death for COVID-19 patients, preventing or weakening cytokine storm could reduce the overall mortality of the disease. Theoretically, corticosteroid therapy might have benefit for severe COVID-19 patients when cytokine storm occurs, as it can exhibit immunosuppressive effects through inhibiting NF-κB signaling; inhibiting the synthesis of proinflammatory cytokines including IL-1β, IL-2, IL-6, TNF-α, IL-17, and granulocyte-macrophage colony-stimulating factor; and reducing the proliferation, activation, differentiation, and survival of T cells and macrophages (18, 41). However, whether corticosteroids are beneficial in the treatment of COVID-19 is controversial, based on the evidence of SARS and Middle East respiratory syndrome (MERS) studies showing that corticosteroids would increase mortality and delay the clearance of coronavirus (42, 43). Recently, one study shows optimistic results, indicating that dexamethasone therapy results in lower 28-day mortality among those who were receiving either invasive mechanical ventilation or oxygen alone (40). In general, the dose, duration, and especially the timing of treatment are the key factors to determine whether corticosteroid therapy is beneficial for COVID-19.

It is noteworthy that impaired lymphocyte function accompanying increased expression of exhaustion markers has been reported in severe COVID-19 patients (44, 45). However, we emphasize that although the number of lymphocytes remains low in severe patients, their function is inconsistent in different phases of illness, which is decided by the time after onset. In line with previous findings, impaired T-cell function was commonly noted in the late phase of severe patients, whereas activation marker expressed on T cells in patients regardless of severity was rapidly increased in the early phase of the disease (46). Thus, the dynamic monitoring of immune responses is of great importance for understanding the pathogenesis of the disease. In addition, a previous study has demonstrated that IL-6 may be the key regulator for the exhaustion of lymphocytes in severe patients (45). Given that the expansion of M-MDSCs was earlier than the increase of IL-6 and was maintained at a high level throughout the illness in patients with poor outcomes, we, therefore, put forward that sustained expansion of M-MDSCs is also one of the key factors contributing to the exhaustion of adaptive immunity. These data suggest that M-MDSCs could be used as a prominent marker for predicting COVID-19 prognosis.

Thus, monitoring the immune status of COVID-19 patients is not only helpful for predicting the prognosis of disease but also improves the therapeutic effect. Consistent with this notion, previous studies have shown that the reduction in inflammation parameters and improvement in lymphopenia are common manifestations of effective treatment (47, 48). Given that we have drawn the dynamic immunological parameter landscape in COVID-19 patients with different outcomes, these immune indicators could be used to guide treatment strategies. For instance, the high inflammatory status provided the theoretical basis of using steroids for the treatment of severe SARS-CoV-2 infection. Furthermore, when machine learning was used to evaluate the combined application of multiple indicators, it would further improve the prediction effect and simplify the work of clinicians (49).

Several limitations of the study should be mentioned. First, this was a single-center study that was limited by the small sample size. Further validation by other centers with a large sample size is required to confirm the predictive role of inflammation markers. Second, we did not classify some cell subsets accurately. For instance, lymphoid DCs were characterized as CD3^−^CD19^−^CD14^−^CD56^−^HLA-DR^+^ cells in CD45^+^ lymphocytes. However, we did not classify different types of DCs, such as CD123^+^ DCs, CD11c^+^ DCs, CD14^+^ DCs, and CD141^+^ DCs. Moreover, several other types of cells such as MDSCs and innate lymphoid cells display similar phenotypes in comparison with DCs, which could cause bias to the results of DCs. Third, given that the number of MDSCs in several types of malignancy was increased in peripheral blood, the effect of MDSC on predicting COVID-19 prognosis in patients who had malignancy would be affected.

This study has described the landscape of immune responses throughout COVID-19 illness, highlighting the importance of host immunity in patients with COVID-19.

## Data Availability Statement

The original contributions presented in the study are included in the article/**Supplementary Material**. Further inquiries can be directed to the corresponding authors.

## Ethics Statement

The studies involving human participants were reviewed and approved by the ethical committee of Tongji Hospital, Tongji Medical College, Huazhong University of Science and Technology, Wuhan, China (IRB ID: TJ-C20200128). The patients/participants provided their written informed consent to participate in this study.

## Author Contributions

FW, GT, SW, ZX, HH, and ZS designed the study. FW, MH, HH, YL, GT, WL, QL, and LM performed experiments. SW, ZX, and HH collected the clinical information and classified the patients. FW, MH, and ZS performed the statistical analysis and drafted the manuscript. All authors contributed to the article and approved the submitted version.

## Funding

This study was supported in part by grants from the National Mega Project on Major Infectious Disease Prevention (2017ZX10103005-007).

## Conflict of Interest

The authors declare that the research was conducted in the absence of any commercial or financial relationships that could be construed as a potential conflict of interest.

## Publisher’s Note

All claims expressed in this article are solely those of the authors and do not necessarily represent those of their affiliated organizations, or those of the publisher, the editors and the reviewers. Any product that may be evaluated in this article, or claim that may be made by its manufacturer, is not guaranteed or endorsed by the publisher.

## Supplementary Material

Supplementary Figure 1 Gating strategies of HLA-DR^+^CD4^+^ T cells, HLA-DR^+^CD8^+^ T cells, and CD45RO^+^CD4^+^ T cells.

Supplementary Figure 2 Gating strategies of CD27^+^CD38^-^ memory B cells and CD27^+^CD38^high^ plasma cells.

Supplementary Figure 3 Gating strategies of CD16^+^CD14^+^ monocytes (non-classical monocytes) and HLA-DR^-^CD14^+^ M-MDSCs.

Supplementary Figure 4 Gating strategies of lymphoid-derived DCs and CD86^+^ lymphoid-derived DCs.

Supplementary Figure 5 ROC analysis of CD16^+^CD14^+^ monocytes, CD8^+^ T cells, neutrophils, IL-6 and M-MDSCs for distinguishing deceased patients from other patients.
