# Supplementary figures and images for: The Dynamic Immunological Parameter Landscape in Coronavirus Disease 2019 Patients With Different Outcomes
Source: Front Immunol. 2021 Oct 29;12:697622. doi: 10.3389/fimmu.2021.697622 (PMC8586656; doi:10.3389/fimmu.2021.697622)

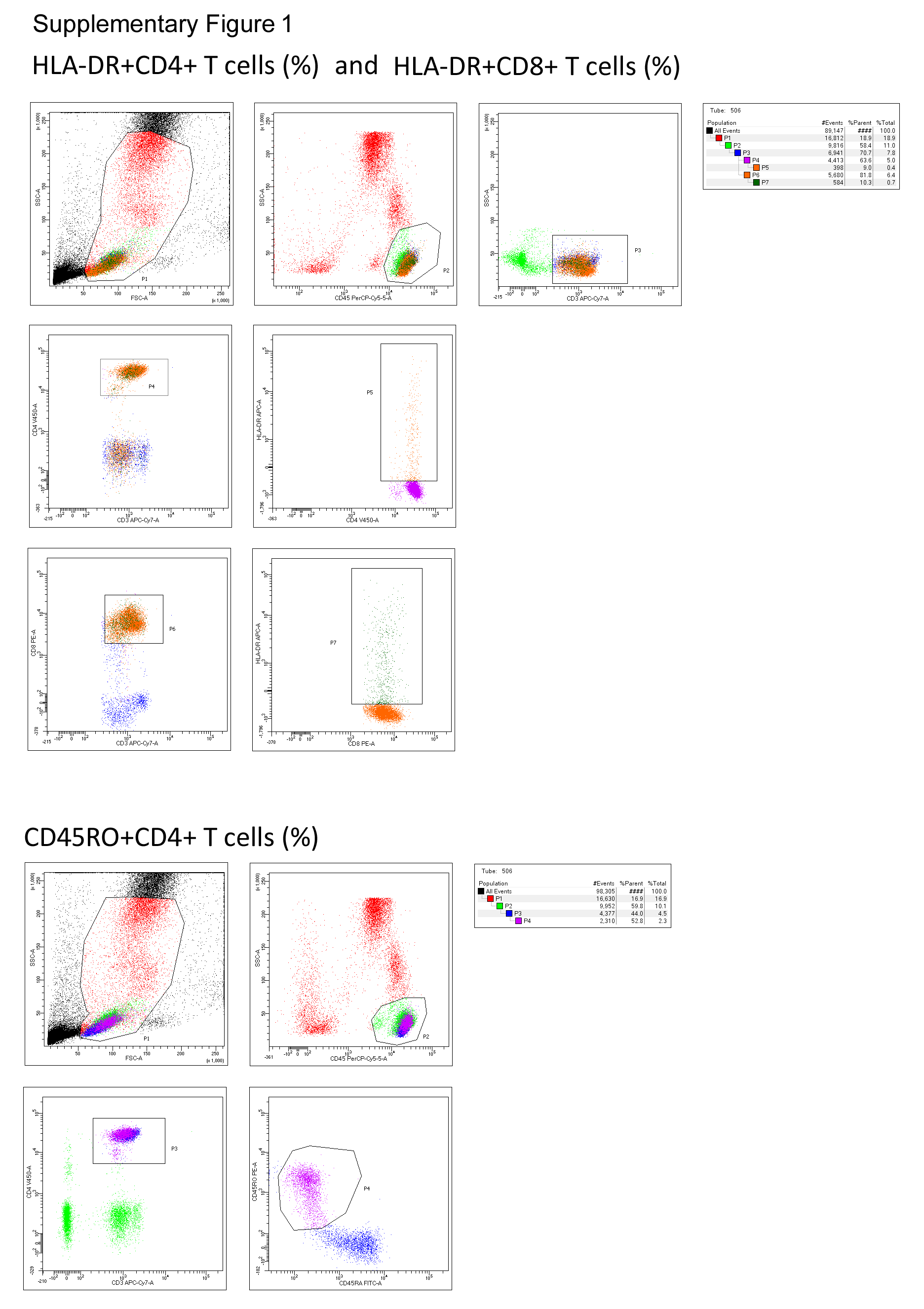

Supplement: Supplementary Figure 1 — Gating strategies of HLA-DR+CD4+ T cells, HLA-DR+CD8+ T cells, and CD45RO+CD4+ T cells. [file Image_1.tif]

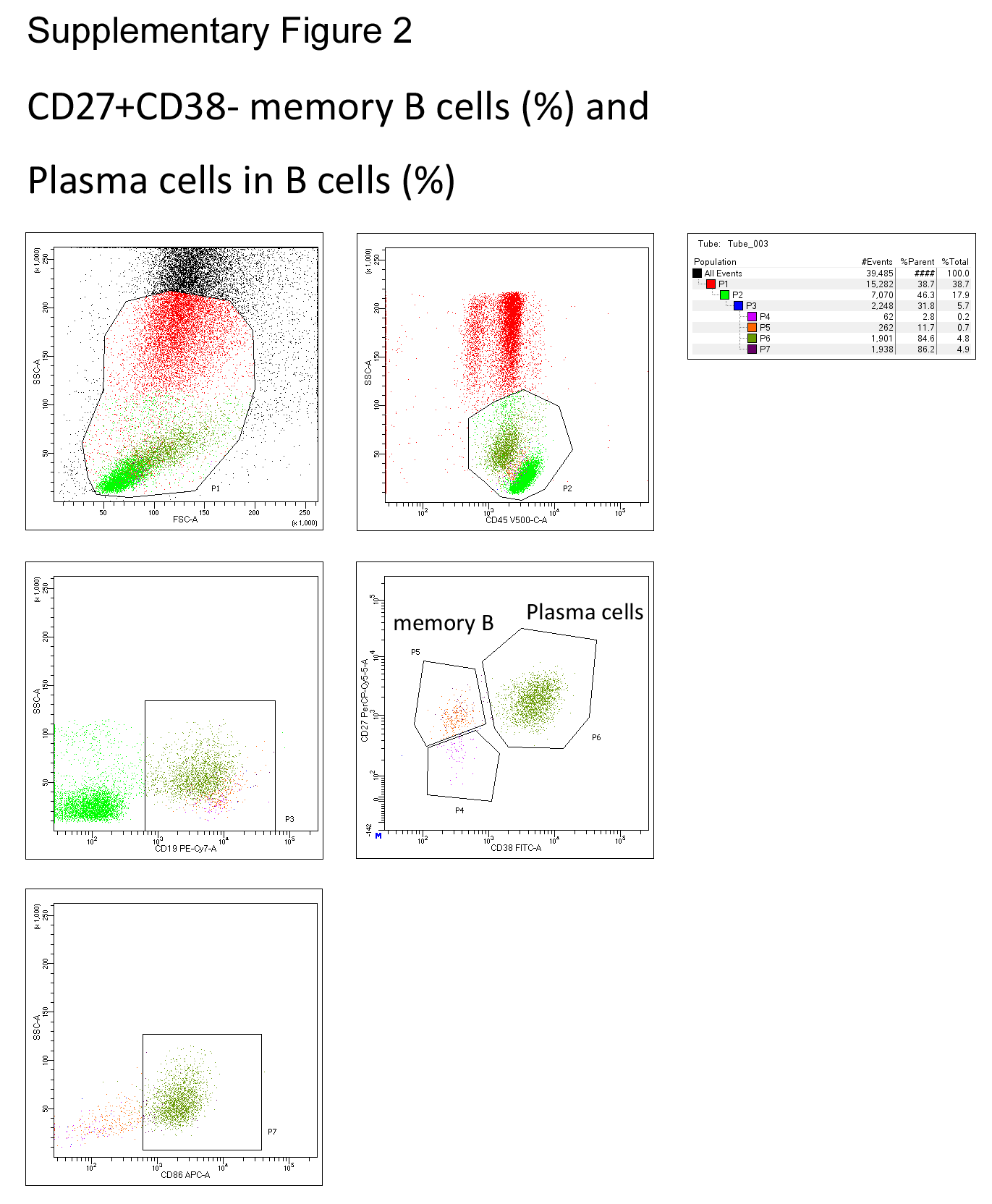

Supplement: Supplementary Figure 2 — Gating strategies of CD27+CD38- memory B cells and CD27+CD38high plasma cells. [file Image_2.tif]

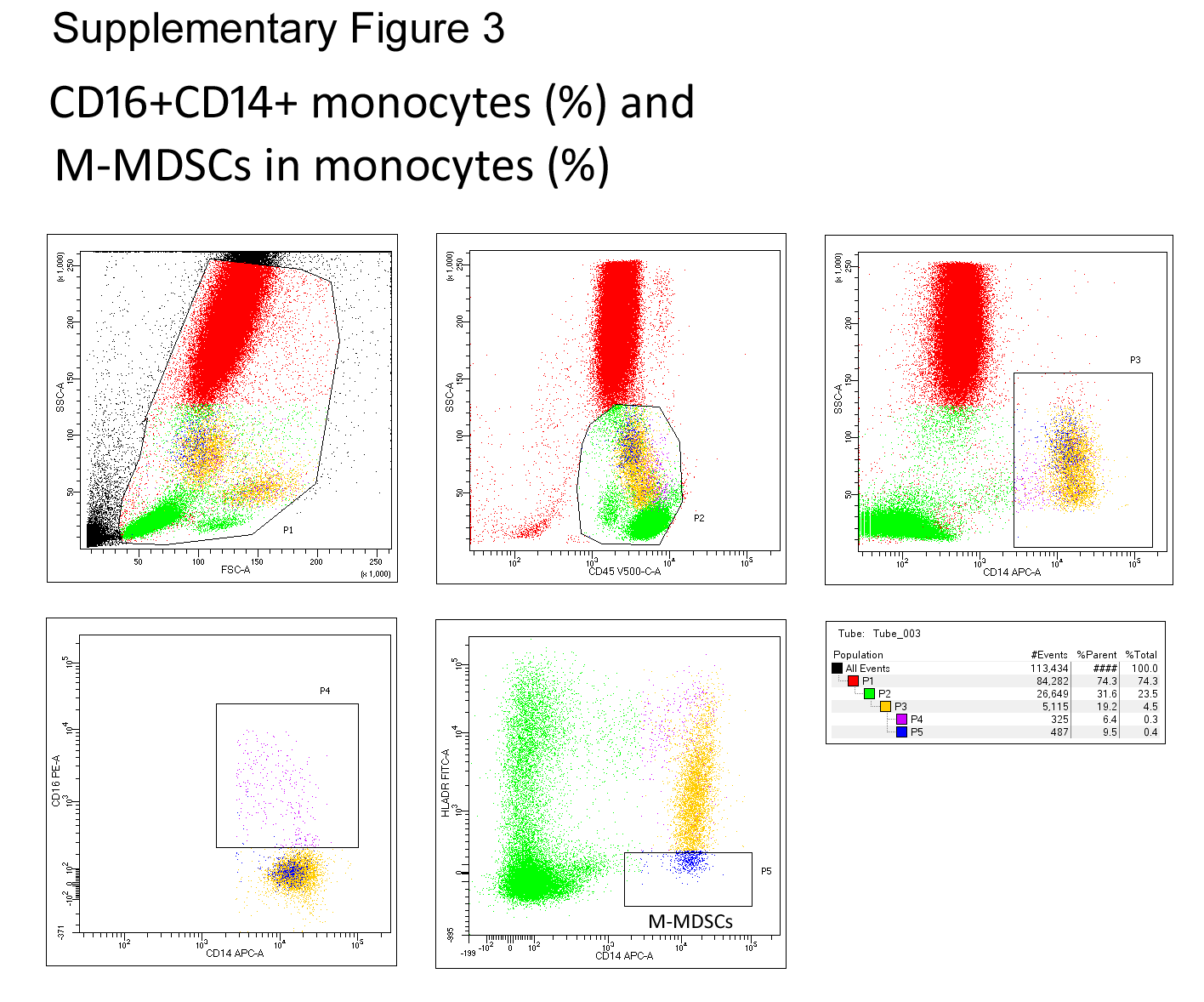

Supplement: Supplementary Figure 3 — Gating strategies of CD16+CD14+ monocytes (non-classical monocytes) and HLA-DR-CD14+ M-MDSCs. [file Image_3.tif]

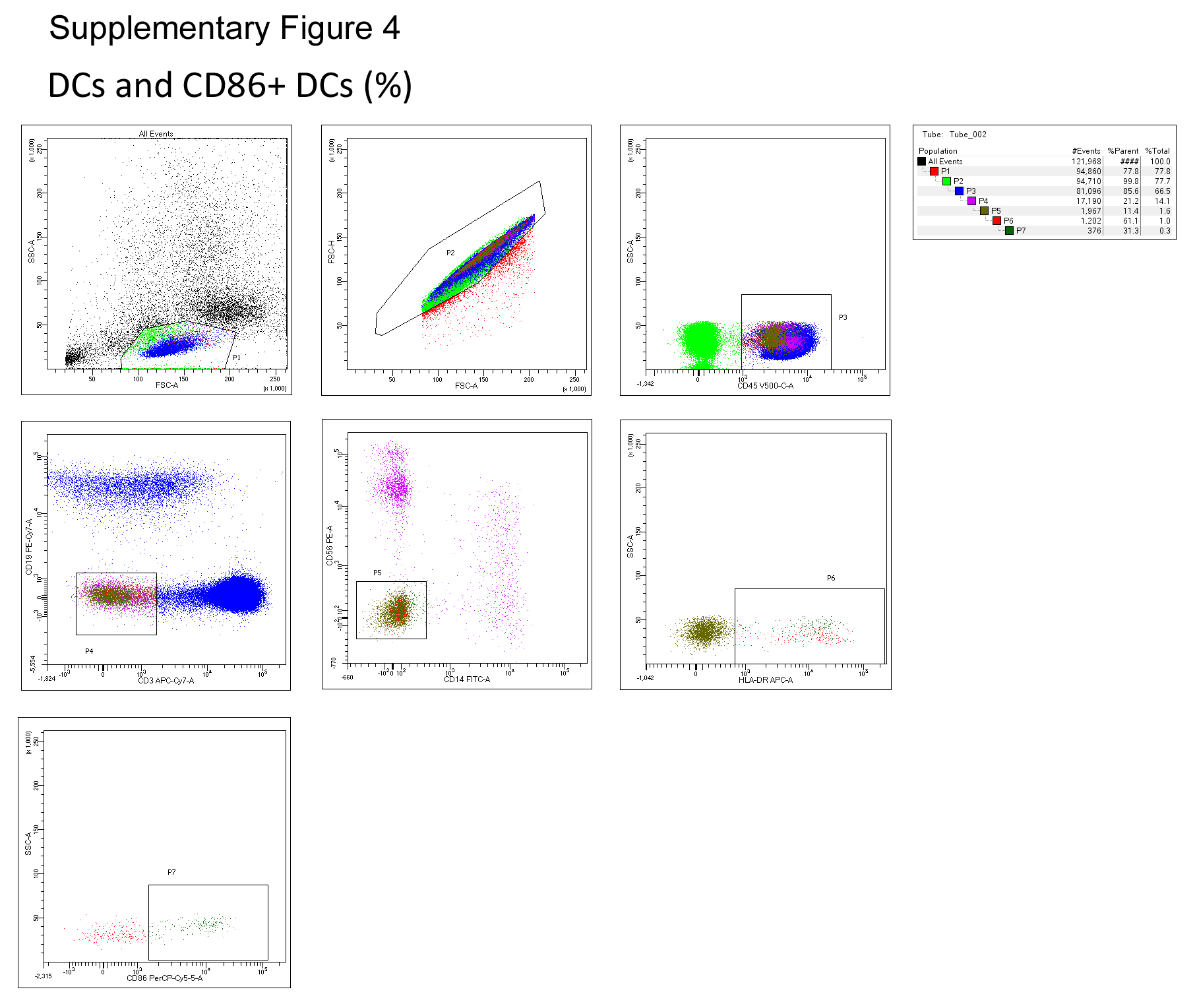

Supplement: Supplementary Figure 4 — Gating strategies of lymphoid-derived DCs and CD86+ lymphoid-derived DCs. [file Image_4.tif]

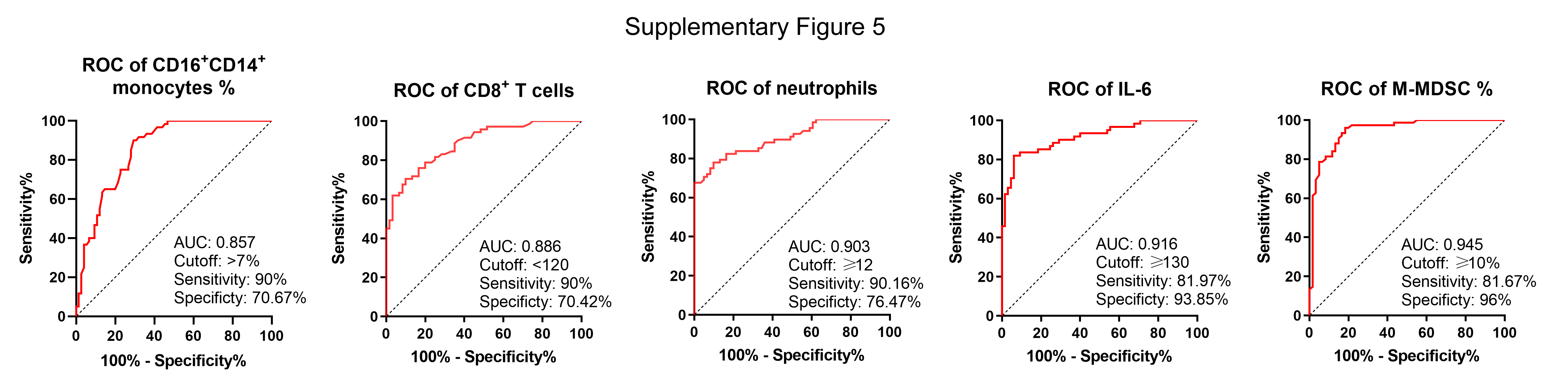

Supplement: Supplementary Figure 5 — ROC analysis of CD16+CD14+ monocytes, CD8+ T cells, neutrophils, IL-6 and M-MDSCs for distinguishing deceased patients from other patients. [file Image_5.tif]
